# Malignant epithelioid angiomyolipoma of the kidney with pulmonary metastases and *p53* gene mutation

**DOI:** 10.1186/1477-7819-10-213

**Published:** 2012-10-08

**Authors:** Jun Li, Ming Zhu, Yan-Li Wang

**Affiliations:** 1 Department of Pathology, The First Affiliated Hospital, Medical College, Zhejiang University, 79 Qingchun Road, Hangzhou, Zhejiang, 310003, China; 2 Department of Head and Neck, Zhengjiang Tumor Hospital, Hangzhou, Zhejiang, 310021, China

**Keywords:** Kidney, Angiomyolipoma, Epithelioid, Malignant, Metastases, *p53* gene mutation

## Abstract

Angiomyolipoma (AML) is a rare tumor mainly arising in the kidney. Here we report the case of a 55-year-old woman with malignant epithelioid angiomyolipoma with *p53* gene mutation. After 7 years from radical nephrectomy of the left kidney, the patient developed multiple lung metastases that showed morphologic features overlapping those of the previously lesion, which was misdiagnosed as renal cell carcinoma. Both renal and pulmonary tumors were reevaluated by immunohistochemical assay, which were showed positive for HMB-45 and p53 protein (95%), but negative for epithelial markers and S-100 protein. A correct diagnosis of malignant epithelioid angiomyolipoma was made on the basis of those results. Meanwhile exon 8 mutation of *p53* gene was detected in the renal tumor by microdissection-PCR-SSCP and sequencing technique indicating that *p53* gene mutation may play an important role in malignant transformation. The patient was died of respiratory failure after 15 years’ follow-up. This is the second report of renal malignant angiomyolipoma with *p53* gene mutation.

## Background

Angiomyolipoma (AML) is now recognized as a clonal mesenchymal neoplasm which stains strongly for melanoma-associated markers (HMB-45). This tumor most commonly arises in the kidney. Although typical renal AML is a benign lesion, few cases of epithelioid AML (EAML) develop malignant clinical courses. To the best of our knowledge, only about 20 cases of renal epithelioid AML with distant metastases have been reported in the English-language literature [1].

The *p53* gene is a tumor suppressor gene that plays an important role in the regulation of cell growth, and the expression of p53 protein has been closely correlated with the presence of *p53* gene mutation. Although a case of renal EAML with a *p53* missense mutation has been reported [2], the pathogenesis and mechanisms of the malignant transformation of AML remain unclear. In the current study, we confirmed mutation of the *p53* gene of malignant renal EAML with pulmonary metastases in a patient.

## Case presentation

**Figure 1:**
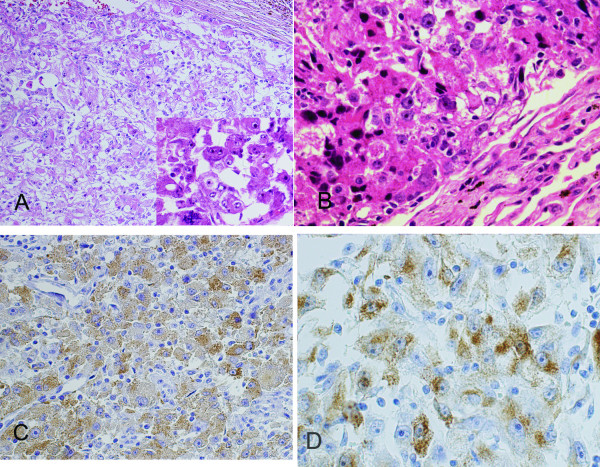
**(A) Microscopically, the renal tumor is composed of polygonal epithelioid cells with abundant eosinophilic cytoplasm, pleomorphic nuclear and hyperchromatic nuclei (hematoxylin and eosin, original magnification 20×), and scattered tumor giant cells and mitotic figures are frequently seen (lower right corner; hematoxylin and eosin, original magnification 40×).** (**B**) The morphologic features of lung relapses overlapped those of the renal lesion (hematoxylin and eosin, original magnification 20×). (**C**) and (**D**) Immunohistochemically, the tumor cells of renal and pulmonary lesions show HMB-45 cytoplasmic reaction. (**C**) DAB, 20×. (**D**) DAB, 40×.

**Figure 2:**
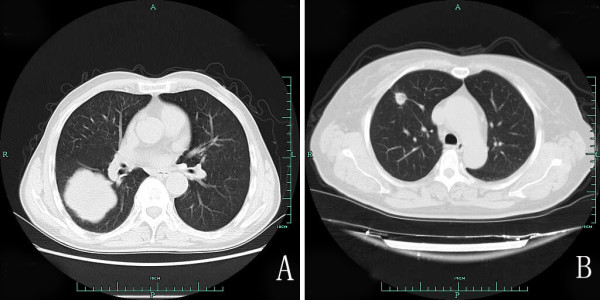
CT scan shows a mass of 8.0 cm in the lower pole (A) and a mass of 1.5 cm in the upper pole of the right lung (B).

**Figure 3:**
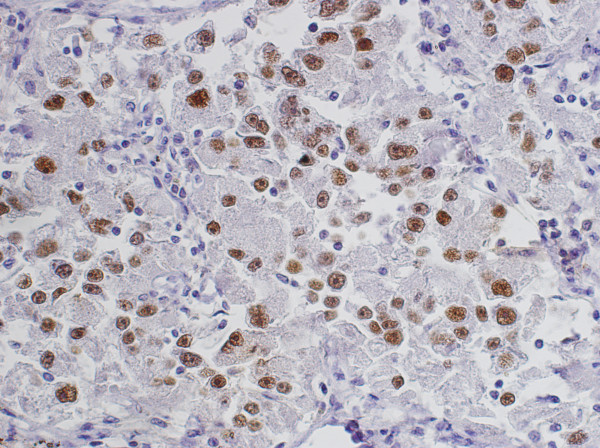
The tumor cells show strong immunoreactivity to p53 protein (3,3′-diaminobenzidine (DAB), original magnification 20×).

**Figure 4:**
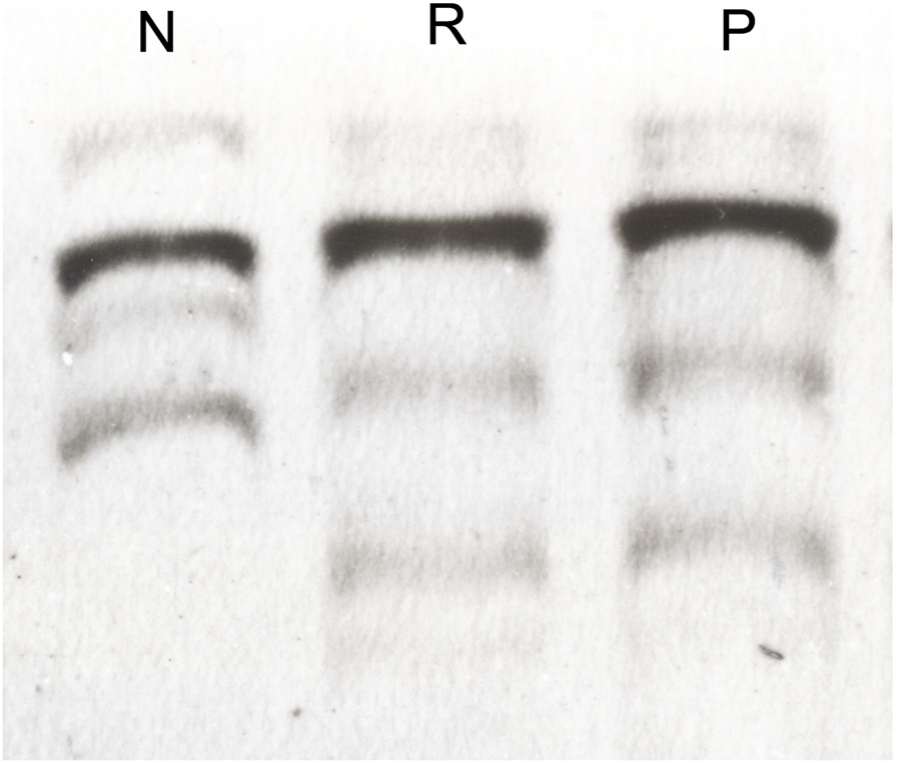
**PCR-SSCP analysis of exon 8 in the *p53 *gene.** Bands shift backward in both renal and pulmonary epithelioid cells. N: normal renal parenchyma as a normal control, R: renal EAML, P: pulmonary EAML.

**Figure 5:**
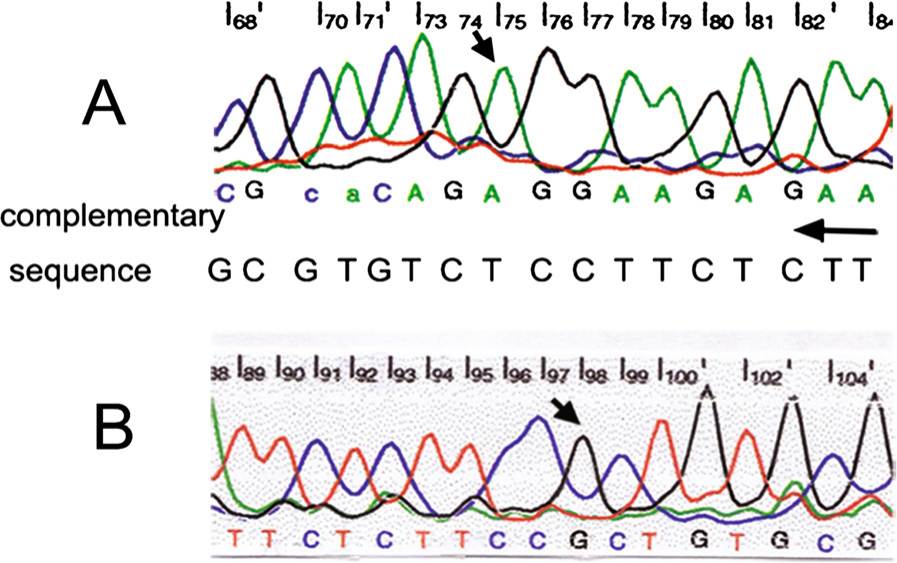
**Direct DNA sequencing of exon 8 of the *p53 *gene.** (**A**) Normal renal parenchyma shows complementary sequence of normal sequence that is promulgated in GenBank. (**B**) The malignant epithelioid component of renal lesion shows T → G transversion at codon 281

A 55-year-old woman was hospitalized with left flank pain for 3 months. Ultrasonography revealed a 7.5-cm solid mass in the left kidney, and radical nephrectomy was performed in May 1994. No evidence of tuberous sclerosis complex or tumors in other locations were found. Macroscopically, the tumor was reddish brown and lobulated, had areas of necrosis and hemorrhaging and a nonencapsulated mass measuring 7.5 cm × 7.0 cm. Microscopically, the tumor was composed of polygonal epithelioid cells in a sheet pattern, which had huge, pleomorphic and hyperchromatic nuclei with abundant eosinophilic cytoplasm. Scattered tumor giant cells, mitotic figures and necrosis were frequently seen (Figure 1A). The diagnosis of renal cell carcinoma (RCC) was made without immunohistochemical examination. About 7 years after nephrectomy, CT showed a mass of 8.0 cm in the lower pole (Figure 2A) and 1.5 cm in the upper pole of the right lung (Figure 2B). Lobectomy of the right lung was performed. The morphologic features of lung relapsed and overlapped those of the renal lesion (Figure 1B). Both renal and pulmonary tumors were reevaluated by immunohistochemical assays. The results showed that the tumor cells of both specimens were positive for vimentin, HMB-45 (Figures 1C and 1D) and p53 protein (approximately 95%) (Figure 3), but they were negative for cytokeratin and S-100 protein. The corrected diagnosis of malignant renal EAML with pulmonary metastases was made. No chemotherapy or radiotherapy was administered postoperatively. The patient died as a result of respiratory failure due to multiple pulmonary metastases about 15 years after nephrectomy. Mutations of exons 5 through 8 were performed in the *p53* gene by the polymerase chain reaction single-strand conformation polymorphism (PCR-SSCP) analysis and sequencing technique according to previously described methods [2]. The sequences of the primers and the condition of amplification are listed in Table 1. The electrophoretic backward-shifted bands were detected in exon 8 (Figure 4) of the *p53* gene in both renal and pulmonary epithelioid cells, but no shift of electrophoretic mobility was found in exons 5, 6 and 7 by PCR-SSCP analysis. Later, sequencing confirmed exon 8 mutation of the *p53* gene, which was a missense mutation of T → G transversion at codon 281 (Figure 5) substituting serine for alanine.

**Table 1:** **The primers of*p53*gene and the condition of amplification**

**Exon**	**Amplified length (bp)**	**Primer sequence (5′-3′)**	**Condition of amplification**
5	183	TACTCCCTGCCCCTCAACAAG	94°C, 1 minute; 55°C, 1 minute; 72°C,
		ATCGCTATCTGAGCAGCGCTC	1 minute; 48 cycles
6	114	GGTCTGGCCCCTCCTCAGCAT	94°C, 1 minute; 63°C, 1 minute;72°C,
		CTCAGGCGGCTCATAGGGCAC	1 minute; 50 cycles
7	111	GTTGGCTCTGACTGTACCACC	94°C, 1 minute; 55°C, 1 minute;72°C,
		CCTGGAGTCTTCCAGTGTGAT	1 minute; 48 cycles
8	135	TGGTAATCTACTGGGACTGAA TCGCTTAGTGCTCCCTGGGGG	94°C, 1 minute; 55°C, 1 minute; 72°C, 1 minute; 48 cycles

## Discussion

AML, one of the members of the PEComa family (tumors showing perivascular epithelioid cell differentiation), arises mainly in the kidney but may also occur in other sites [3,4]. The typical AML is regarded as a benign lesion, despite the presence of nuclear pleomorphism and mitotic activity [2]. Although AML sometimes shows extension into the vena cava or localization in the regional lymph node, it is still considered to be a multifocal lesion rather than metastasis [5]. EAMLs are rare variants with the capability of recurrence and metastasis. Among them, approximately 50% of reported patients developed metastases and 33% of patients died as a result of the disease [1,2,5-9].

There are no specific features of EAML in terms of clinical manifestations and imaging modalities. The appearance of the lesions is similar to the findings in patients with RCC [10]. Moreover, microscopically, the presence of epithelioid cells can result in difficulty in differentiating this tumor from RCC. In our case, the renal tumor was misdiagnosed as RCC without immunohistochemical results initially. In this situation, the immunohistochemical assay is significant in the differential diagnosis. AML is characteristically positive for HMB-45, a fact that is useful for discriminating carcinoma from carcinoma-like AML. In the present case, by retrospective analysis, the tumor cells of renal and pulmonary lesions showed strong immunoreactivity to HMB-45, but neither epithelial markers nor S-100 protein was immunopositive, which allowed a definitive diagnosis of EAML.

The criteria for predicting malignancy in EAML are not well-recognized because of the rarity of this entity. These criteria include associated tuberous sclerosis complex or concurrent AML, tumor size, extrarenal extension and/or renal vein involvement, carcinoma-like growth pattern, cellular atypia, pleomorphic nuclei mitotic activity, and necrosis [1,11]. However, not all cases with cytologic atypia correlate with poor prognosis. Some investigators [7,8] have concluded that aggressive behavior, such as cellular atypia, venous invasion and local recurrence after incomplete surgical resection, should not be interpreted as malignancy, and the diagnosis of malignancy can be made only on the basis of the presence of metastases. In our case, nuclear atypia, mitotic activity and necrosis were palpable, and the diagnosis of malignant EAML was confirmed by the pulmonary metastases.

The mechanism of the malignant transformation of epithelioid AML is not evident. Although *p53* mutation has been described in one case report of renal EAML by Kawaguchi *et al*. [2], no significant *p53* mutation was found in two other cases reported by Ma *et al*. [12] and Sato *et al*. [13]. In the present case, diffuse nuclear p53 immunoreactivity and *p53* gene abnormalities were identified in the renal and pulmonary lesions, which indicate that *p53* mutation may play an important role in the malignant nature of EAML. To the best of our knowledge, this is the second published report of renal malignant AML with *p53* gene mutation.

According to scattered reports, the disease-specific survival for malignant EAML varies greatly (3 months to 9 years) [1,2,5-11], and distant metastases may result in death because effective chemotherapeutic methods of the tumor have not yet been clearly established. In our case, metastatic lesions were demonstrated and directly related to the death of the patient 15 years after nephrectomy.

Because of the rarity of malignant EAML, the optimal treatment is uncertain. Definitive diagnosis and radical tumor surgical resection at an early stage could be important in the treatment of patients with renal EAML. Adjuvant radiation and chemotherapy may have some beneficial effect on overall survival, but the effects need further investigation.

## Conclusions

We report one rare case of a patient with renal malignant EAML with pulmonary metastases and *p53* gene mutation. This entity is difficult to distinguish from RCC preoperatively. Accurate diagnosis depends on morphologic, immunohistochemical examination and clinical follow-up. *p53* gene mutation might play a role in the malignant transformation.

## Consent

Written informed consent was obtained from the next-of-kin of the patient for publication of this case report and any accompanying images. A copy of the written consent is available for review by the Editor-in-Chief of this journal.

## Competing interests

The authors declare that they have no competing interests.

## Authors’ contributions

J LI, and YL Wang conceived the case report, participated in drafting the manuscript and conducted critical review. M Zhu took part in assembling data and participated in writing the manuscript. All authors read and approved the final manuscript.
